# Numerical Simulation of Temperature Fields during Laser Welding–Brazing of Al/Ti Plates

**DOI:** 10.3390/ma16062258

**Published:** 2023-03-11

**Authors:** Mária Behúlová, Eva Babalová

**Affiliations:** Faculty of Materials Science and Technology in Trnava, Slovak University of Technology in Bratislava, Ulica Jána Bottu 2781/25, 917 24 Trnava, Slovakia; eva.babalova@stuba.sk

**Keywords:** laser welding–brazing, Al/Ti joint, welding parameters, numerical simulation, temperature field, microstructure, tensile strength

## Abstract

The formation of dissimilar weld joints, including Al/Ti joints, is an area of research supported by the need for weight reduction and corrosion resistance in automotive, aircraft, aeronautic, and other industries. Depending on the cooling rates and chemical composition, rapid solidification of Al/Ti alloys during laser welding can lead to the development of different metastable phases and the formation of brittle intermetallic compounds (IMCs). The effort to successfully join aluminum to titanium alloys is associated with demands to minimize the thickness of brittle IMC zones by selecting appropriate welding parameters or applying suitable filler materials. The paper is focused on the numerical simulation of the laser welding–brazing of 2.0 mm thick titanium Grade 2 and EN AW5083 aluminum alloy plates using 5087 aluminum filler wire. The developed simulation model was used to study the impact of laser welding–brazing parameters (laser power, welding speed, and laser beam offset) on the transient temperature fields and weld-pool characteristics. The results of numerical simulations were compared with temperatures measured during the laser welding–brazing of Al/Ti plates using a TruDisk 4002 disk laser, and macrostructural and microstructural analyses, and weld tensile strength measurements, were conducted. The ultimate tensile strength (UTS) of welded–brazed joints increases with an increase in the laser beam offset to the Al side and with an increase in welding speed. The highest UTS values at the level of 220 MPa and 245 MPa were measured for joints produced at a laser power of 1.8 kW along with a welding speed of 30 mm·s^−1^ and a laser beam offset of 300 μm and 460 μm, respectively. When increasing the laser power to 2 kW, the UTS decreased. The results exhibited that the tensile strength of Al/Ti welded–brazed joints was dependent, regardless of the welding parameters, on the amount of melted Ti Grade 2, which, during rapid solidification, determines the thickness and morphology of the IMC layer. A simple formula was proposed to predict the tensile strength of welded–brazed joints using the computed cross-sectional Ti weld metal area.

## 1. Introduction

Due to the demand for lightweight structures and the reduction in fuel consumption, especially in the aerospace and automotive industries, the development of joining technologies, for dissimilar aluminum–titanium components, has been one of the main focuses in welding research for several years [1,2,3,4,5,6]. The principal advantages of such joints are the combination of properties of both materials, including the low density, high specific modulus, good formability and fracture toughness of aluminum alloys, and the excellent corrosion properties, high tensile strength, and high-temperature resistance of titanium alloys. On the other hand, joining aluminum to titanium alloys using conventional fusion welding processes is associated with a number of real problems, due to the significant differences in the alloys in regard to thermophysical properties (melting point, heat conductivity, thermal expansion, etc.), their limited mutual solubility, the intermixing of the melted phases and, in particular, the development of brittle intermetallic phases during solidification of the weld pool [7,8,9,10].

Generally, the interface layers with intermetallic compounds (IMCs), such as TiAl, TiAl_2_, TiAl_3_ and Ti_3_Al, cause brittleness and a decrease in mechanical properties of the Al/Ti weld joints. The IMC layers represent mostly the sides of the initiation and propagation of the weld cracks. Therefore, to produce sound joints of Al/Ti alloys, which are characterized by increased toughness and strength, the intermetallic phase layer should be limited to minimum thickness [11,12,13,14,15,16,17,18,19,20,21]. Therefore, various weld configurations and different welding methods have been tested to join aluminum to titanium alloys, including arc technologies—TIG [22,23] or MIG welding [24], CMT [25], laser and electron beam welding [26,27,28,29,30,31], resistance spot welding [32], friction welding [33,34], FSW [35,36,37,38,39], explosion welding [40,41,42,43], diffusion welding [44,45,46], and ultrasonic welding [47,48,49,50], and also some hybrid technologies, such as laser–TIG/laser–MIG welding [51,52,53,54], and laser or ultrasonic vibration-assisted brazing [55,56,57].

The application of numerical simulations to welding processes offers numerous advantages for studying and explaining the physical principles of complex phenomena related to joining processes [58,59,60,61,62]. The results of numerical simulations can be used for a time-saving and cost-effective assessment of the impact of welding parameters on the quality of Al/Ti dissimilar weld joints, and the setting of optimal parameters of the welding process [63,64,65,66,67,68,69,70,71].

Casalino et al. [63] investigated the process of laser welding of AA5754 and Ti6Al4V sheets in butt joint configuration using laser shifting to the Ti side. For numerical simulations in ANSYS finite element code, they exploited a thermo–mechanical model to predict not only the thermal cycles and the temperature fields, but also the thermo–mechanical behavior and shape of the weld pool. By comparing the numerical and experimental results, they found that the seam quality and formation of IMC brittle interface depended on the laser offset and the linear energy of the laser source. The increased amount of molten titanium resulted in inhomogeneity of the IMC layer and promoted the initiation and propagation of cracks.

A simple model and ANSYS commercial code were applied by Casalino et al. [64] to predict the seam morphology and to improve the main thermal aspects involved in the welding process of 2 mm thick AA5754 aluminum alloy and T40 commercially pure titanium sheets. They modeled the heat input to the weld using a constant heat generation rate to the nodes of selected elements, supposing V-shaped and X-shaped beads. The thermal contact resistance between the Al and Ti sheets at the weld centerline was taken into account and calibrated, along with the heat generation rate, in order to achieve a good agreement between the experimental and numerical results.

The FEM simulations of the thermal cycles during Al/Ti fiber offset welding [65] showed that the 3D Gaussian heat source model provided better approximations of heat fluxes and cooling rates during laser welding than the 2D Gaussian heat source model. These parameters are crucial to predict the direction of grain growth in the process of rapid solidification of a weld pool.

A 3D Gaussian ellipsoidal volumetric heat source model was used for numerical simulation of temperature fields in laser beam welding of Al/Ti sheets of different thicknesses [66]. To analyze the formation of welded–brazed Al/Ti butt joints using a filler wire, a simulation model, incorporating the 3D conical heat source model, was suggested [67,68].

Zhan et al. [69] compared the results of numerical simulation of temperature fields performed using a 3D finite element model and a combined Gaussian heat source model with the experimental grain morphology of weld joints developed during laser welding–brazing of 1050 aluminum alloy to Ti–6Al–4V. The surface heat source was adopted to model the influence of the laser on the surface of the welded sample, while the Gaussian rotating body heat source simulated the keyhole effect.

Metallurgical joining of dissimilar Ti–6Al–4V and Al–Mg–Si alloys was achieved successfully using a new re-melting process [70], which effectively improved the tensile strength of the joints. The joining method consisted of the welding–brazing process with the laser beam focusing on the Al sheet, followed by an additional remelting pass formed by the laser beam shifting to the Ti sheet. To support an explanation of the microstructure evolution, an FEM simulation was conducted. A hybrid heat source model, consisting of a Gauss surface heat source and a Gaussian damped volume heat source, was applied to obtain a precise temperature distribution.

Zhou et al. [71] investigated the temperature and thermal stress distribution in laser lap welding of Ti6Al4V and 2024-T4 alloys using the coupled thermal–mechanical finite element model and a composite heat source model consisted of a double ellipsoid heat source and a Gaussian rotating body heat source. The microstructural evolution and microhardness of the lap joints were investigated in detail on the basis of the simulation results. The comparison of the obtained results revealed a close correlation between the thermal cycle process and the growth of grains in both the heat-affected zone and the fusion zone.

More comprehensive coupled fluid–thermal or fluid–thermal–mechanical models were used to analyze the processes of laser welding of aluminum alloys [72,73], titanium alloys [74], steels [75,76,77,78] or various dissimilar joints, especially a combination of different steel grades [79,80] or Al, Mg, Ti, Cu and Fe alloys [81,82,83,84,85,86,87,88]. 

A critical review of experimental and modeling studies on laser welding is provided in [89]. The authors focused their attention on the effect of process parameters on the geometry, thermodynamics, fluid dynamics, microstructure, and porosity characteristics of the melt-pool, with the goal of developing a methodology for verifying and validating models and results in future melt-pool modeling studies. The methodology of characterization of the developed IMC layers in the processes of joining dissimilar materials is presented, for example, in [11,12,16,20,70,90,91,92].

The presented contribution is focused on the modeling and numerical simulation of temperature fields in the process of laser beam welding–brazing of Ti Grade 2 and EN AW5083-H111 aluminum alloys using the ANSYS finite element code. The developed simulation model of the joining of dissimilar Al/Ti materials was used for computational experiments and investigation of the impact of an applied laser power, laser beam offset, and welding speed on the temperature fields in the welded–brazed plates. Based on the results obtained from the simulations, suitable welding parameters were proposed to conduct physical experiments of laser welding–brazing of dissimilar sheets made of Al/Ti alloys using the TruDisk 4002 disk laser (TRUMPF SE+Co., KG, Ditzingen, Germany) and to find correlations between welding parameters and mechanical properties of the welded–brazed joints.

## 2. Material and Methods

Laser beam welding was applied to produce welded–brazed butt joints of two plates with dimensions of 50 mm × 100 mm × 2 mm from dissimilar materials: commercially pure titanium Grade 2 and EN AW5083-H111 aluminum alloy with the chemical compositions given in Table 1 and Table 2, respectively. The KD 7000 Fronius filler wire feed unit was exploited in the joining process. As a filler material, filler wire, with a diameter of 1.2 mm made of 5087 aluminum alloy (Table 3), was used. 

The wire feed rate was 260 cm·min^−1^. The angle between the filler wire and the base metal was 15°. Argon, at a flow rate of 18 L·min^−1^, was applied as a shielding gas to protect the weld bead and weld root during laser beam welding.

Based on previous research [67,93,94], different welding parameters were considered for the production of the welded–brazed butt joints to investigate the effect of laser power, welding speed and laser beam offset on the temperature fields in the welding–brazing process:

- laser power from 1.6 kW to 2.0 kW,
- welding speed from 25 mm·s^−1^ to 30 mm·s^−1^ and
- laser beam offset from 200 μm to 460 μm from the weld centerline towards the aluminum sheet.

The impact of selected technological parameters of a welding–brazing process on the quality of Al/Ti joints was examined using both numerical simulations in ANSYS v18.2 software and experimental methods. Table 4 lists the welding parameters applied for the experimental preparation of welded–brazed joints using the TruDisk 4002 continuous wave disk laser. The maximum power of this laser equipment was 2.0 kW, the wavelength was 1.03 μm, and the beam quality parameter (BPP) was 8 mm·mrad. A laser light cable with a core diameter of 400 μm was used to transport the laser beam to the focusing optics BEO D70 mounted on a Fanuc M710iC/50 6-axis robot. The disk laser beam was focused at +2 mm above the surface of the welded plates. Samples with dimensions of 50 mm × 50 mm × 2.0 mm, prepared by cutting, were polished and cleaned with acetone prior to welding to ensure good surface contact in the weld centerline. 

Two K-type thermocouples were employed to measure the temperatures during the experiments. One thermocouple was placed on the top surface of the Ti plate, while the other was positioned on the bottom side of the Al plate. Both thermocouples were located 3 mm away from the weld centerline. The results of the experimental temperature measurements were utilized to verify the simulation model and computed temperatures.

The test specimens, made from prepared welded–brazed Al/Ti joints, were subjected to mechanical testing, and macrostructural and microstructural analyses. Transverse tensile tests were performed on a LabTest 5.250 SP1-VM test machine (Labortech Ltd., Praque, Czech Republic) with a maximum loading force of 250 kN, at a crosshead speed of 1 mm·s^−1^.

Specimens for macroscopic and microscopic examination were taken from cross-sections perpendicular to the weld centerline. These specimens were etched using Keller’s etchant with a chemical composition of 2 mL HF, 6 mL HNO_3_, and 92 mL distilled H_2_O for 3–5 s. Macroscopic and microscopic observations were carried out using a ZEISS Stemi 2000-C device with an AxioCam ERc5s camera (Carl Zeiss Microscopy GmbH, Jena, Germany), and NEOPHOT 32 light microscope (Carl Zeiss Microscopy GmbH, Jena, Germany). A JEOL 7600 F scanning electron microscope (SEM), produced by JEOL Ltd., Tokyo, Japan, was used to study the IMC layer developed at the Ti Grade 2 and weld metal interface.

## 3. Theoretical Background and Simulation Model

The numerical simulation of laser welding processes typically involves solving coupled thermal–fluid and stress–strain problems that incorporate phase transformations [59,95]. This comprehensive approach allows for the evaluation of temperature distribution, taking into account the convection of the melt in the weld pool, as well as the dimensional changes and deformations of the weldments. It also enables the prediction of stresses that arise during the welding process and residual stresses remaining in welds after their cooling to room temperature. These predictions can be used to adjust and optimize welding parameters in order to eliminate potential weld cracking and failure.

In this study, the initial numerical simulations of the laser welding–brazing process were specifically focused on analyzing the temperature fields, with the objective of determining appropriate welding parameters for experimental testing of the laser welding process and for predicting the quality of welded–brazed joints. 

The evolution of transient temperature fields arising during the laser welding–brazing process can be obtained by solution of the heat diffusion equation [96]

(1) $$\rho c_{p}\frac{\partial T}{\partial t}=\left[\frac{\partial T}{\partial x}\left(\lambda_{x}\frac{\partial T}{\partial x}\right)+\frac{\partial T}{\partial y}\left(\lambda_{y}\frac{\partial T}{\partial y}\right)+\frac{\partial T}{\partial z}\left(\lambda_{z}\frac{\partial T}{\partial z}\right)\right]+q_{v}$$

where *T*(*x, y, z, t*) is the temperature [°C, K], *t* is the time [s], *ρ* is the density [kg·m^−1^], *c*_p_ is the specific heat [J·kg^−1^·K^−1^], *λ*_x_, *λ*_y_, *λ*_z_ are the thermal conductivities [W·m^−1^·K^−1^] in the *x*, *y*, *z* directions of the Cartesian coordinate system and *q*_v_ is the volumetric density of internal heat sources [W·m^−1^], i.e., the heat generated in unit volume per unit time. Solving the partial differential Equation (1) requires specifying the geometric, physical, initial, and boundary conditions. This includes the definition of geometric shapes and dimensions of the structures to be welded, their material properties, and initial temperatures at time *t* = 0 s. The heat extraction from the welded components to the surroundings is taken into account by the boundary conditions. In addition, the heat input to the weld, due to the method of heating applied in fusion welding, must be specified using appropriate boundary conditions or internal heat sources.

**Figure 1:**
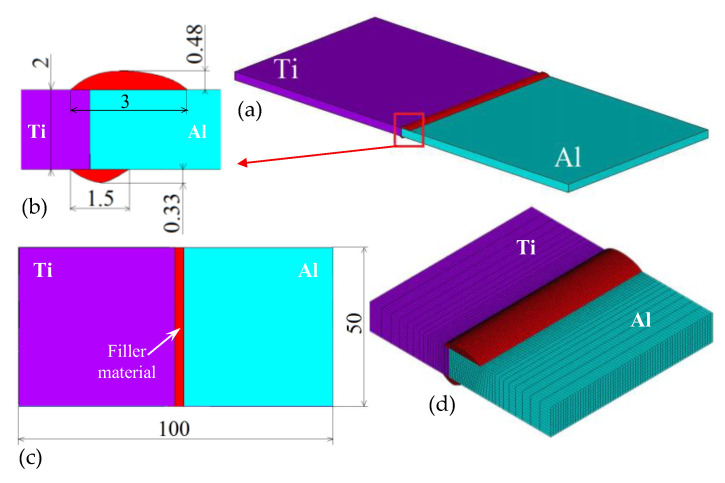
Geometric model (**a**) with the dimensions in front view (**b**) and top view (**c**) and a detail of the generated FE mesh (**d**).

Taking into account the definition of the problem, the dimensions of the experimental welded plates were 50 mm × 50 mm × 2.0 mm. The weld joint geometry, including the filler material (Figure 1a), was designed based on previous experiments [68,97]. The dimensions of the weld joint and the welded samples are shown in Figure 1b (front view) and Figure 1c (top view). The 3D finite element mesh was generated in the ANSYS software [98] using the element type of SOLID70, based on the expected temperature gradients. The mesh density was highest in the weld pool region and the heat-affected zone. The finest mesh density of 0.005 mm was in a direction perpendicular to the weld line. The element length in the welding direction was kept constant at 0.2 mm. Detail of the generated mesh is shown in Figure 1d.

**Figure 2:**
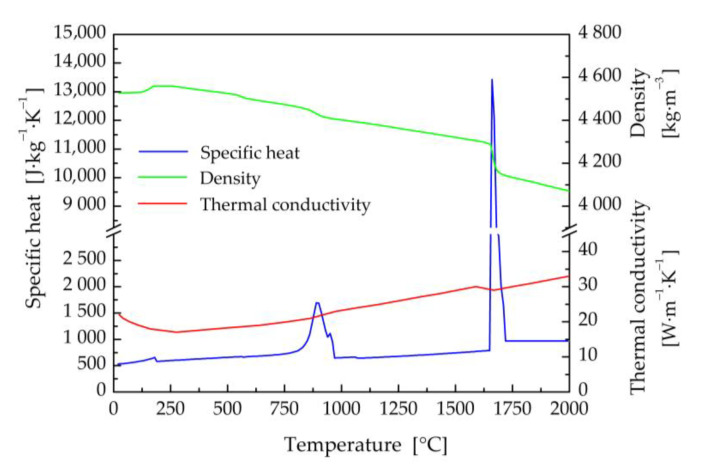
Thermal properties of Ti Grade 2 in dependence on temperature.

**Figure 3:**
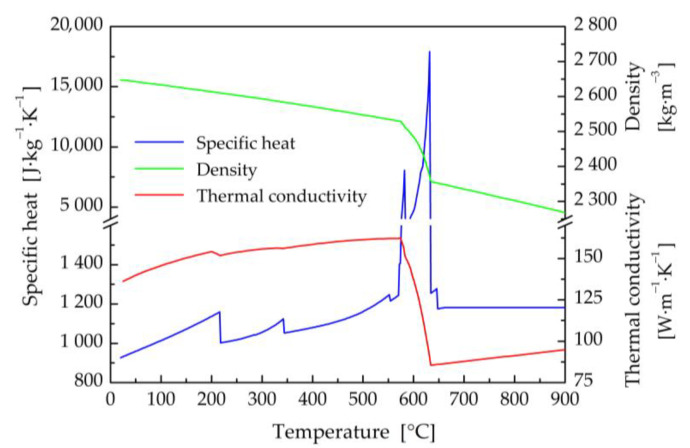
Thermal properties of the EN AW5083-H111 alloy in dependence on temperature.

**Figure 4:**
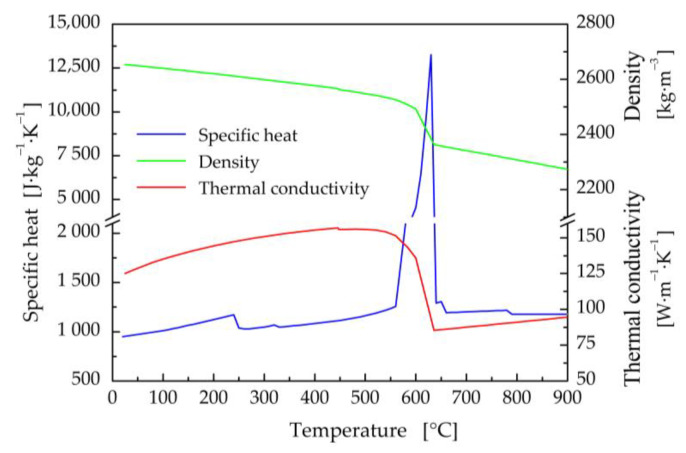
Thermal properties of the filler material 5087 in dependence on temperature.

The thermophysical properties (thermal conductivity, specific heat, and density) of titanium and aluminum base materials, and filler wire in their dependence on the temperature (Figure 2, Figure 3 and Figure 4) were calculated using JMatPro v6.1 software [99]. Titanium Grade 2 represents commercially pure titanium with a purity of 99.2% by weight. It has a solidus temperature of *T*_S_ = 1665 °C and a liquidus temperature of *T*_L_ = 1713 °C. The equilibrium solidus and liquidus temperatures for the EN AW5083-H111 aluminum alloy were calculated to be 575 °C and 635 °C, respectively. The solidus and liquidus temperatures for the filler material were similar, with values of *T*_S_ = 573 °C and *T*_L_ = 635 °C. 

The method of modified specific heat was exploited to take into account the enthalpy of fusion for all considered materials [100]. The temperature dependences of the specific heat capacity in Figure 2, Figure 3 and Figure 4 also include transformations in the solid state, e.g., α ↔ β phase transformation in titanium Grade 2.

Mathematically, the initial condition can be expressed as follows:

*T*_0_ = *T*(*x, y, z, t =* 0), (2)

where *T*_0_ is the initial temperature of the welded components, which is typically assumed to be equal to the ambient temperature. In our case, the supposed initial temperature of the sheets to be welded was 20 °C.

The boundary condition of the third kind [96] was employed to define the cooling of the welded plates. This boundary condition makes it possible to define the removal of heat from the surface of the welded sheets to the surroundings not only by convection, but also by the mechanism of radiation. The heat removal by radiation is proportional to the fourth power of the surface temperature of the cooled body and becomes the dominant method of heat transfer at higher temperatures. Using this approach to define the boundary conditions is more efficient from a numerical perspective and results in shorter computation times.

Mathematically, the boundary condition of the third kind, which describes the cooling of the plate (sample) by convection and radiation to the argon shielding gas and surrounding air, can be expressed as follows:

(3) $$-\lambda \;\;gradT=h\left(T_{w}-T_{f}\right)$$

where *T*_w_ is the surface temperature [°C, K^−1^], *T*_f_ is the surrounding fluid temperature [°C, K^−1^] and *h* represents the combined heat transfer coefficient [W·m^−1^·K^−1^] given by the sum of the convection heat transfer coefficient *h*_C_ [W·m^−1^·K^−1^] and the radiation heat transfer coefficient *h*_R_ [W·m^−1^·K^−1^]

(4) $$h=h_{C}+h_{R}=h_{C}+\frac{\varepsilon \;\sigma_{0}(T_{w}^{4}-T_{f}^{4})}{T_{w}-T_{f}}$$

where *ε* [-] is the emissivity of the materials depending on the surface temperature and *σ*_0_ is the Stefan–Boltzmann constant (*σ*_0_ = 5.67040 × 10^−8^ W·m^−1^·K^−1^). The values of the convection heat transfer coefficient for the top and bottom surfaces of the air-cooled welded plates were calculated in the dependence on the surface temperature using classical convection heat transfer correlations [96]

(5) $$h_{C}=\frac{\lambda_{g}Nu}{L}=\frac{\lambda_{g}CRy^{n}}{L}$$

where $Nu=\frac{h_{C}L}{\lambda_{g}}$ is the Nusselt number [-], $Ry=\frac{gL^{3}\alpha_{v}\Delta T}{av}$ is the Rayleigh number [-], *L* denotes the characteristic length [m], *λ*_g_ is the thermal conductivity of the gas [W·m^−1^·K^−1^], *g* is the gravitational acceleration [m·s^−2^], *α*_v_ is the thermal expansion coefficient [K^−1^], *ν* is the kinematic viscosity [m^−1^·s^−1^], *a* is the thermal diffusivity of the gas [m^−1^·s^−1^] and Δ*T* corresponds to the temperature difference between the surface temperature and the surrounding temperature (Δ*T = |T*_w_ − *T*_f_|). Parameters *C* and *n* depend on the value of the Rayleigh number: *C* = 1.18 and *n* = 1/8 for 10^−3^ < *Ry* ≤ 500, *C* = 0.54 and *n* = 1/4 for 500 < *Ry* ≤ 2 × 10^7^ and *C* = 0.135 and *n* = 1/3 for 2 × 10^7^ < *Ry* ≤ 1 × 10^13^ [96].

**Figure 5:**
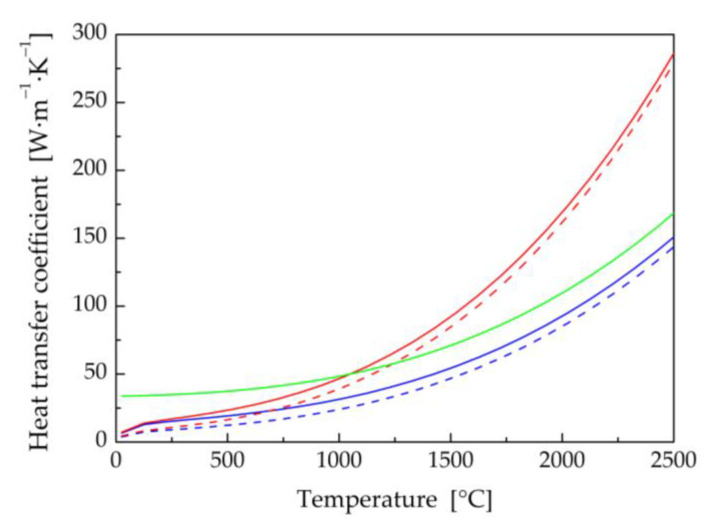
Calculated values of the combined heat transfer coefficient in dependence on the surface temperature of the welded sheets and the filler material.

The computed values of the convection heat transfer coefficient *h*_C_ for plate cooling by air-free convection were multiplied by 1.3 for the top sheet surfaces and by 0.7 for the bottom sheet surfaces, respectively [96]. However, the convection heat transfer coefficient *h*_C_ is much lower at higher temperatures compared to the radiation heat transfer coefficient, which is independent of the location of the cooled surface. The relationship between the combined heat transfer coefficient and surface temperature for the top and bottom Al and Ti plate surfaces is shown in Figure 5. The values of the heat transfer coefficient for cooling the Ti plate were higher than those for the Al plate because titanium has a higher emissivity compared to aluminum alloy.

In the zone of filler material, cooling by forced convection and radiation was considered (Figure 5). At an argon flow rate of 18 L·min^−1^, the convection heat transfer coefficient by forced convection was computed at the level of 33.2 W·m^−1^·K^−1^. At higher temperatures, the effect of radiation was again more conspicuous.

The thermal resistances between the Al–Ti plates, between the Ti sheet and the filler material, and also between the Al sheet and the filler material were neglected, as their influences on the transient temperature fields during laser welding were not so important as, for example, in the case of solid-state welding using the FSW method [101]. 

Different methods can be used to model the heat input in fusion welding, e.g., moving-point heat sources, line heat sources, surface or volumetric heat sources [102,103,104,105,106,107,108,109,110,111,112,113,114]. Application of temperatures on the top surface of a weld pool (boundary condition of the first kind) or temperatures to the volume of the molten zone [115] represent the easiest and fastest ways to model a heat source and to calculate the temperature distribution in welding processes. However, this requires an estimation of the weld pool geometry and the weld pool temperatures, which are generally unknown. 

**Figure 6:**
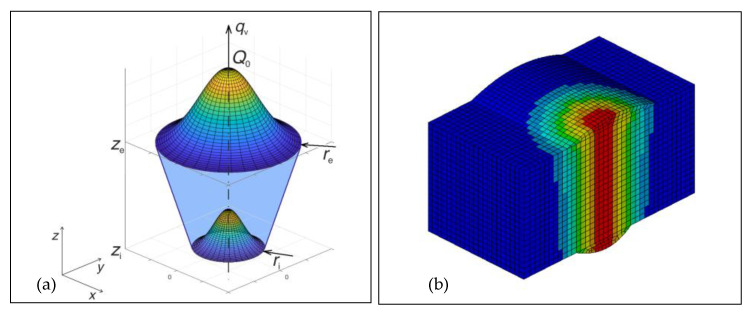
3D conical heat source model: (**a**) scheme and (**b**) an example of its application to the FE model.

In order to conduct a numerical simulation of the laser welding–brazing of dissimilar Ti/Al sheets, a 3D conical volumetric heat source model (Figure 6) was adopted. The heat input to the weld can be described by the function [108]

(6) $$q_{v}(x,y,z)=\frac{9\eta \;Q_{0}\;e^{3}}{\pi (e^{3}-1)}\;\frac{1}{\left(z_{e}-z_{i})(r_{e}{}_{}^{2}+r_{e}r_{i}+r_{i}{}_{}^{2}\right)}\exp\frac{-3(x^{2}+y^{2})}{r_{0}^{2}(z)}$$

$$r_{0}^{}(z)=r_{e}+\frac{r_{i}-r_{e}}{z_{i}-z_{e}}(z-z_{e})$$

where *q*_v_ [W·m^−3^] is the volumetric density of internal heat sources, *Q*_0_ is the maximum intensity (power) of the heat source [W], *η* is the efficiency [-], *r*_e_ and *r*_i_ [m] are the surface radii in the planes, *z* = *z*_e_ and *z* = *z*_i_, respectively, and *x, y,* and *z* represent the instantaneous spatial coordinates of the heat source (Figure 6a). A special subroutine was developed to define the conical volumetric heat source model, which was implemented in the ANSYS software system. 

## 4. Verification of the Simulation Model

To verify the simulation model for the computer simulation and analysis of the welding–brazing of plates of the dissimilar materials, Ti Grade 2 and EN AW5083-H111, temperatures during the laser welding experiments were measured by thermocouples of the K-type located 3 mm away from the weld centerline on the top surface of the Ti plate and on the bottom surface of the Al plate. The placement of the thermocouple on the bottom surface of the aluminum plate was supposed to prevent damage of this thermocouple, as the laser beam was offset towards the Al plate. For temperature recording, a QuantumX MX1609KB (HBM, Darmstadt, Germany) measurement device was used. 

**Figure 7:**
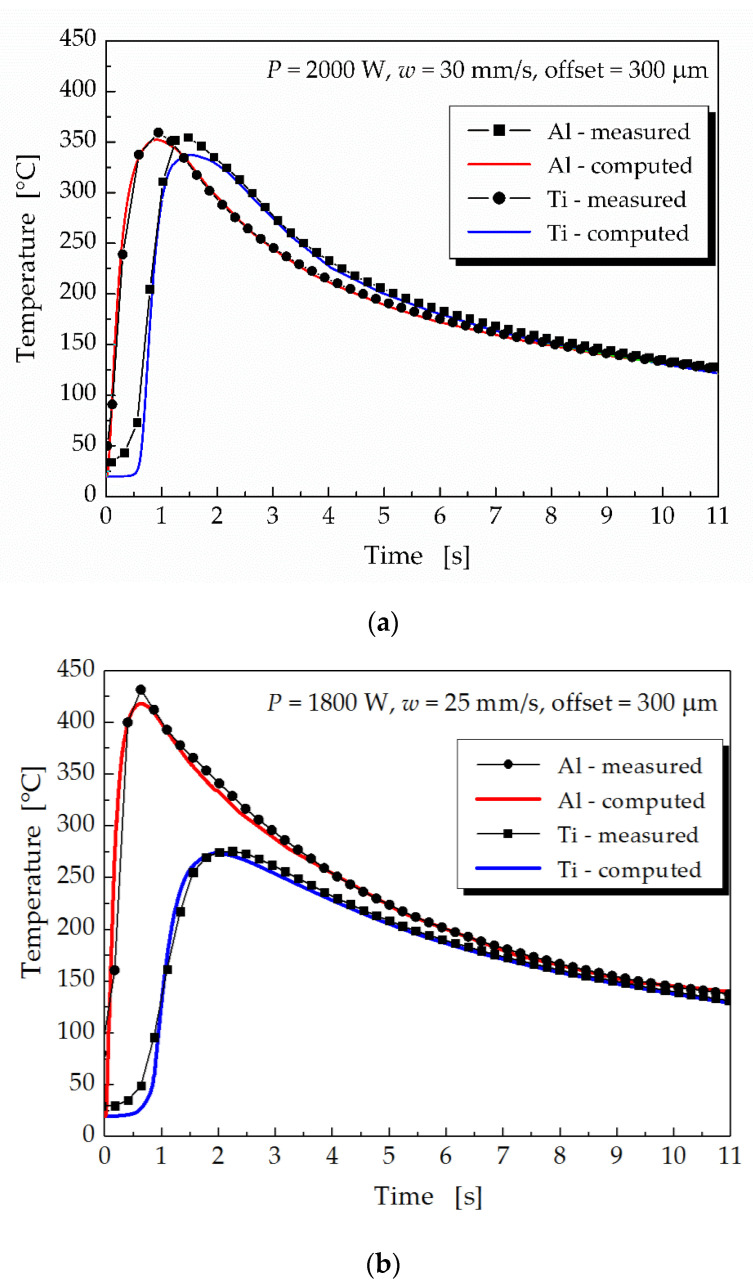
Computed and experimentally measured thermal cycles in welding–brazing (**a**) sample 2 and (**b**) sample 6.

Numerical simulations were performed using the ANSYS software. The time dependences of the computed and measured temperatures during the laser welding of samples 2 and 6 (Table 4) are plotted in Figure 7. Sample 2 was prepared with a laser power of 2000 W, a welding speed of 30 m·s^−1^ and an offset of 300 μm. The parameters for welding of sample 6 were a laser power of 1800 W together with a welding speed of 25 m·s^−1^ and the same offset of 300 μm. The maximum temperature on the bottom side of the Al plate was higher at a laser power of 1800 W and the lower welding speed of 25 m·s^−1^, compared to the maximum temperature at a laser power of 2000 W, but a higher welding speed of 30 m·s^−1^. However, the maximum temperature at the top of Ti plate was higher when higher laser power was applied. The use of higher laser power, combined with a higher welding speed, produced smaller temperature differences in the welded materials.

When comparing the results of the numerical simulations and temperature measurements, the calculated temperatures were slightly lower than the measured ones, as can be seen in Figure 7. The temperature differences were primarily due to the accuracy of the thermocouple positioning and the higher initial/surrounding temperature during the experiments than what was assumed in the numerical simulations. Another possible factor was the heat removal from the base materials to the filler material. The geometry and assigned materials (Figure 1) did not change during the numerical simulation of the welding–brazing process. This meant that the filler material was not deposited continuously but was part of the model during the simulation of the entire welding process. A similar approach to modeling the welding–brazing process was successfully applied by Chen [15]. According to our experience, a numerical simulation of the consecutive addition of filler material in laser welding processes is not as important as in the case of a numerical simulation of arc welding processes, which are carried out with lower welding speeds. 

From the comparison of results obtained through numerical simulations and experimental measurements, it could be inferred that the simulation model developed was sufficiently accurate and could be used to study the effects of welding parameters on the temperature fields generated during the laser welding–brazing of Ti Grade 2 and EN AW5083-H111 sheets using 5087 aluminum alloy filler wire. 

## 5. Results and Discussion

As part of the research on laser welding–brazing of dissimilar Al–Ti materials, 27 computer experiments were conducted using the presented simulation model and the ANSYS software system. The laser beam power varied from 1.6 kW to 1.8 kW and 2.0 kW, the welding speed was supposed to be 25 mm·s^−1^, 28 mm·s^−1^ and 30 mm·s^−1^ and the laser beam offset was set from 200 μm to 300 μm and 460 μm from the weld centerline toward the aluminum side. The obtained results of the performed numerical simulations were used to evaluate the effect of welding parameters, namely, the laser power, the welding speed, and the laser beam offset, on transient temperature fields in the welding–brazing of Ti/Al plates. 

**Figure 8:**
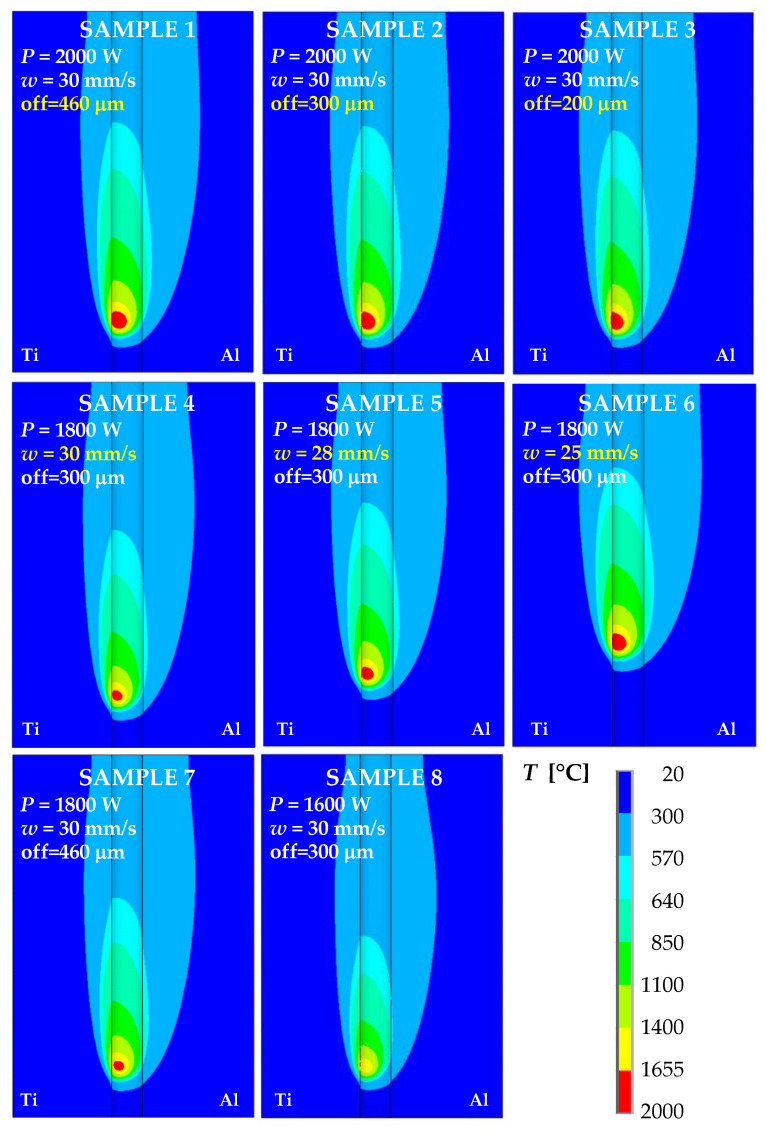
Details of the temperature fields at the time of 1 s for samples 1 to 8 computed using different welding parameters according to Table 4.

As an illustration, the details of the temperature fields calculated at 1 s are shown in Figure 8 for the welding parameters listed in Table 4 and correspond to samples 1 to 8.

Figure 8 confirms the expected asymmetry of the temperature fields in Al and Ti plates, which was mainly caused by the different thermal properties of aluminum and titanium alloys. It could be partly attributed to setting the offset of the laser beam towards the Al plate. In all cases, with the exception of sample 8 prepared with a laser power of 1600 W, the maximum temperatures exceeded the liquidus temperature of both materials being joined. Decreasing the laser beam offset, the maximum temperature location moved towards the titanium plate (samples 1–3). This conclusion was also supported by the comparison of temperature fields at 1800 W laser power and different offsets (samples 4 and 7). The decrease in welding speed for the laser beam power of 1800 W and the laser beam offset of 300 μm led to enlargement of the weld pool dimensions (samples 4–6). These results are analyzed in detail in the following sections. 

**Figure 9:**
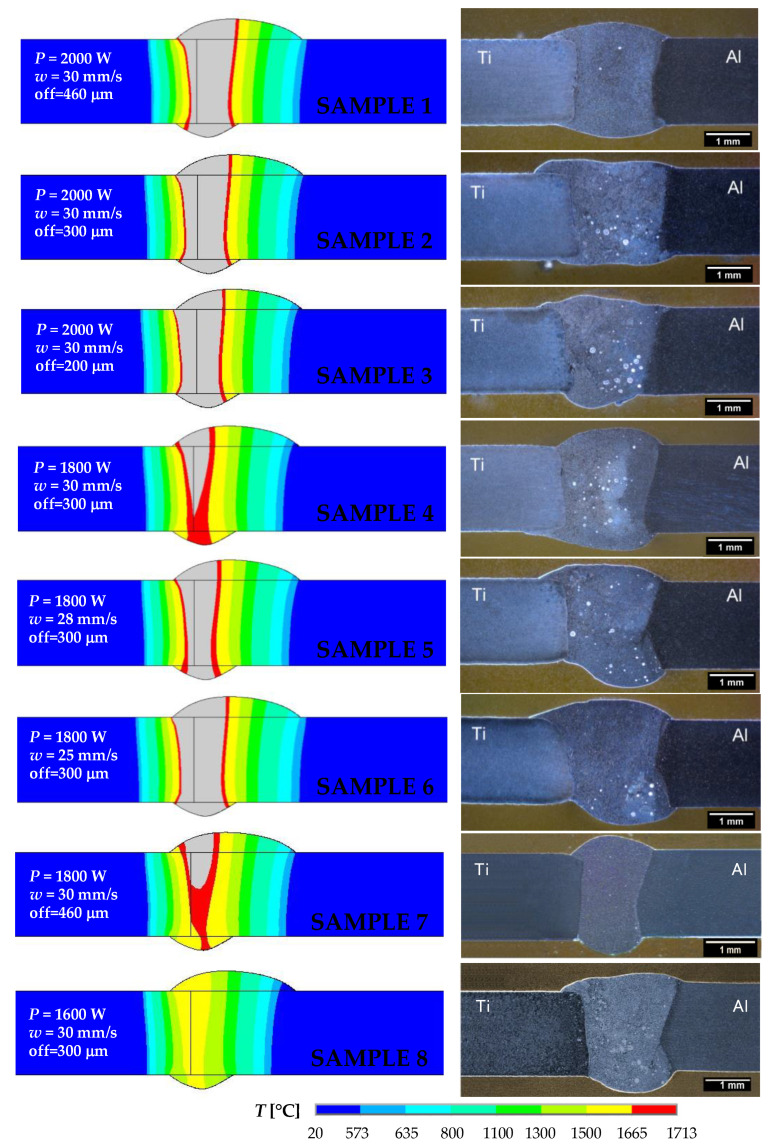
Comparison of the calculated temperature fields in the cross-sections perpendicular to the welding direction with the observed macrostructures of welded–brazed joints produced with various welding parameters according to Table 4.

In Figure 9, temperature fields in the weld cross-sections perpendicular to the welding speed are illustrated, together with the macrostructures of the prepared weld joints for samples 1–8. 

The light blue colour represents the temperature range corresponding to the temperatures between solidus and liquidus for the Al alloy and filler material. Areas at temperatures between solidus and liquidus of Ti Grade 2 are shown in red. The gray regions are above 1713 °C, which is the liquidus temperature of Ti Grade 2. 

**Figure 10:**
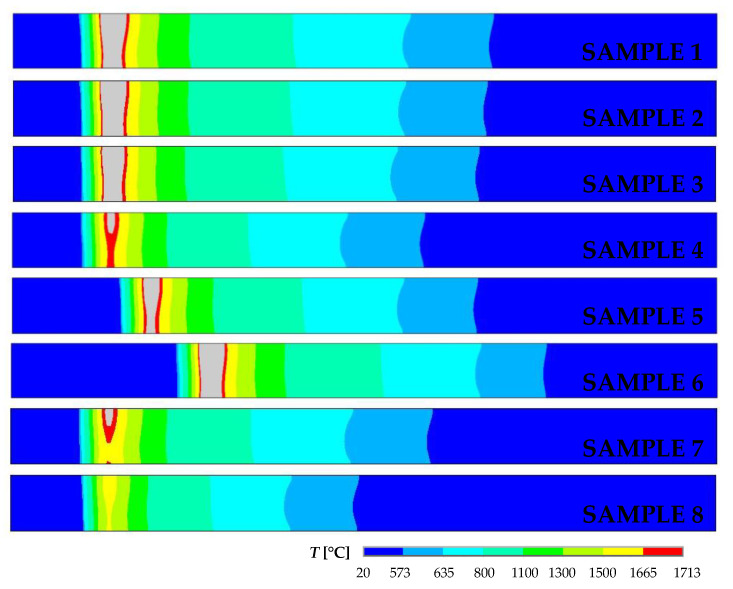
Details of computed temperature fields in the longitudinal sections along the weld centerline in a time of 1 s for samples from 1 to 8.

When the laser power was 1600 W (sample 8), there was no melting of Ti Grade 2. The melted area on the Ti side increased with increasing laser power (sample 4 and sample 2). The effect of laser offset on the melted Ti area is illustrated using samples 1–3 for 2000 W laser power, and also by samples 4 and 7 for 1800 W laser power. Of course, with increasing welding speed at 1800 W laser power and 300 mm laser offset, the size of the melted area on the Ti side decreased (samples 4–6). Figure 10 shows details of the computed temperature fields in the longitudinal sections along the weld centerline at 1 s for samples 1 to 8. This figure also confirmed that, with a laser power of 1600 W, the temperatures of the weld pool were lower than the solidus temperature for Ti Grade 2 (sample 8). The length of the melted zone increased with increasing laser power (samples 1–4 and 7–8) and decreasing welding speed (samples 4–6).

### 5.1. Influence of Laser Beam Offset on the Temperature Fields

**Figure 11:**
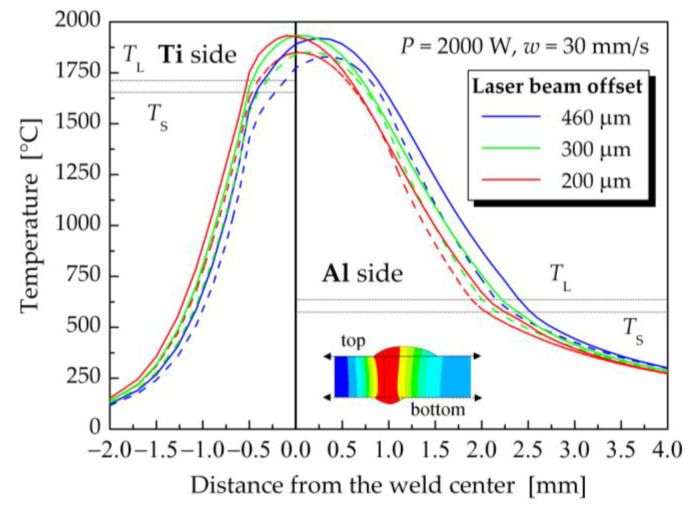
Dependence of the temperature on the distance from the weld centerline for the considered offsets of the laser beam on the top surface (solid lines) and bottom surface (dashed lines) of welded–brazed plates (the laser power of 2000 W and the welding speed of 30 mm·s^−1^).

The influence of laser beam offset on the temperature distribution in welded–brazed materials was studied primarily for a laser power of 2000 W and a welding speed of 30 mm·s^−1^ (samples 1–3). Figure 11 presents a graphical representation of the effect of laser beam offset on the temperature distribution along the lines perpendicular to the weld centerline on the both the top and bottom surfaces of the welded–brazed plates. The laser beam offset had practically no effect on the maximum temperature of the weld pool, which decreased slightly with increasing laser offset. However, it had a more significant influence on the weld pool width on the titanium and also on the aluminum sides. For instance, when the laser beam offset increased from 200 μm to 460 μm, the width of the fusion zone above the liquidus temperature on the top surface on the titanium side decreased from 0.518 mm to 0.342 mm. On the other hand, the same change in the laser beam offset resulted in an increase in the size of the weld pool on the aluminum side from 2.076 mm to 2.406 mm. It could be assumed that a further increase in the laser beam offset might prevent the melting of the Ti plate. This tendency was confirmed by computed temperature fields at a laser power 1800 W (Figure 9 and Figure 10) and offsets of 300 μm (sample 4) and 460 μm (sample 7). 

**Figure 12:**
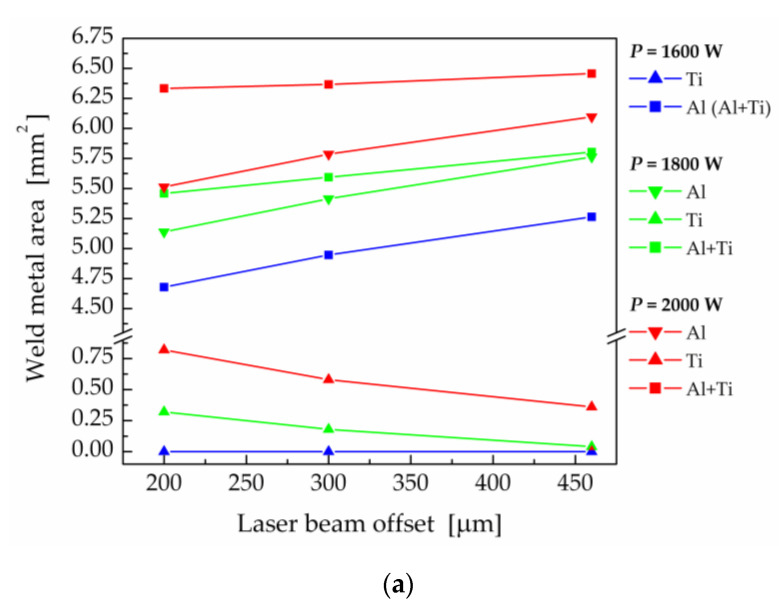

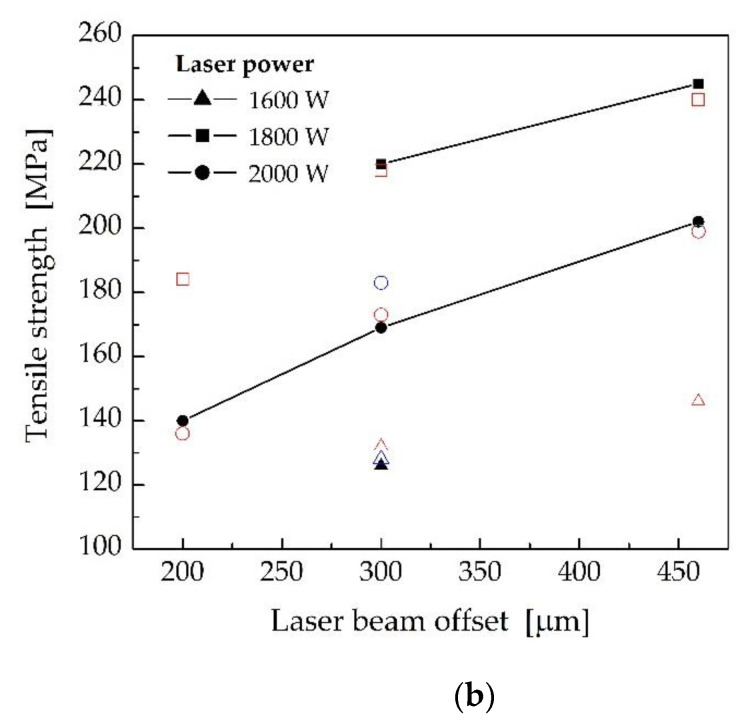
Effect of the laser beam offset (**a**) on the weld metal area and (**b**) on the ultimate tensile strength of the weld joints prepared with different laser powers (red symbols—data according to [93], and blue symbols—data published in [97]).

In the next step, the size of the melted area in the cross-sections perpendicular to the welding directions on the Al and Ti side were evaluated directly using the ANSYS program code. The size of the melted cross-sectional area of the weld joint on the Al side, and on the Ti side, as well as the total melted cross-sectional weld area depending on the laser beam offset for laser powers of 2000 W, 1800 W and 1600 W is plotted in Figure 12a. Figure 12b shows the results of the transversal tensile test performed for samples 1–3, 4, 7 and 8, which were prepared with different laser offsets at various laser powers. The graph also contains the published data [93,97] that were measured for weld joints prepared with similar welding parameters.

At 1600 W laser power, the fusion of Ti Grade 2 did not occur. The calculated size of the melted area on the Al side increased with the increased laser beam offset. The size of the molten area on the Al side is important in terms of the wetting of the Ti plate. The measured ultimate tensile strength (UTS) of the weld joints increased with larger offsets. However, the maximum ultimate tensile strength measured was only at the level of 135 MPa [93], which corresponds to approximately 50% of the UTS of the EN AW5083-H111 base material. For higher laser powers, the size of the melted area on the Ti side decreased with increasing laser beam offset. On the other hand, the melted area on the Al side and also the total melted area (on the Ti + Al sides) increased with increasing laser beam offset. 

At a laser beam power of 2000 W, the ultimate tensile strength of the weld joints increased from 140 MPa for a laser beam offset of 200 μm to 169 MPa at off = 300 μm and to 202 MPa for off = 460 μm. However, the results of the tensile test of weld joints prepared at 2000 W laser power were worse in comparison with the values of ultimate tensile strength measured for weld joints produced at a laser power of 1800 W. The decisive parameter appeared to be the size of the melted zone on the Ti side, which was smaller in the case of lower laser power. As the laser beam offset increased, the ultimate tensile strength of the weld joints increased as well. The maximum ultimate tensile strength was measured for sample 7, produced with a laser power of 1800 W, welding speed of 30 mm.s^−1^ and a laser offset of 460 μm. The ultimate tensile strength reached the value of 245 MPa, which is nearly 90% of the UTS of the EN AW5083-H111 base material. 

These results correspond to previously published conclusions [6,27,90,94], in which offsetting the laser beam on aluminum during butt welding the minimum thickness of the IMC layer and the maximum mechanical properties can be achieved. 

### 5.2. Influence of Laser Power on the Temperature Fields

**Figure 13:**
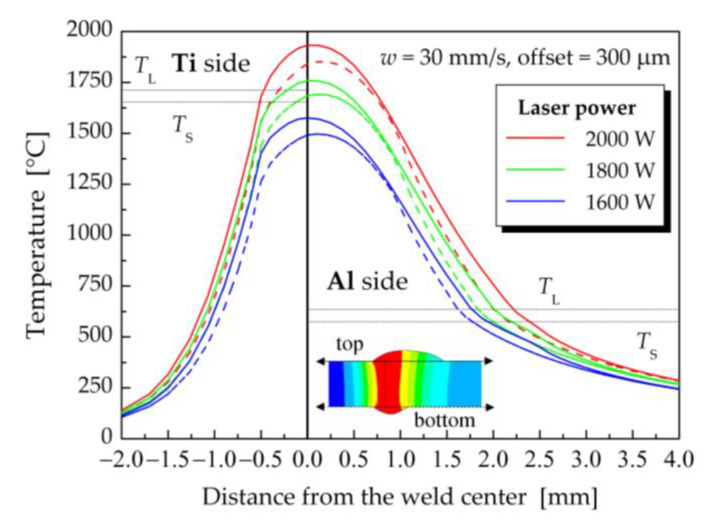
Dependence of temperature on the distance from the weld centerline for selected laser powers on the top surface (solid lines) and bottom surface (dashed lines) of the welded plates (a welding speed of 30 mm·s^−1^ and a laser beam offset of 300 μm).

The influence of laser power on the temperature fields during the Al/Ti dissimilar laser welding process was evaluated for a laser beam offset of 300 μm, a welding speed of 30 mm·s^−1^ and laser powers from 1600 W to 2000 W. Figure 13 shows the dependence of temperature on the distance from the weld centerline on the top and bottom surfaces of the welded–brazed plates for the investigated laser powers. 

As can be seen in Figure 13, the changes in laser power had a more pronounced impact on the calculated temperature fields than did variation in the laser beam offset. In the case of a laser power of 2000 W, the temperatures in the central zone of the weld were higher than the liquidus temperature of Ti Grade 2. The width of the melted area on the top surface of Ti was 0.710 mm, while on the bottom surface of the Ti plate it was 0.665 mm. At a laser power of 1800 W, the centerline temperatures on the top surface reached values above the liquidus temperature for Ti Grade 2, but the temperatures at the bottom weld surface ranged between *T*_S_ and *T*_L_ for Ti Grade 2. This meant that the titanium plate was not fully remelted throughout the thickness. Laser power of 1600 W along with a welding speed of 30 mm·s^−1^ and a laser offset of 300 μm were not enough to melt the Ti sheet. The computed maximum temperatures in the weld centerline were only at a level of 1575 °C.

**Figure 14:**
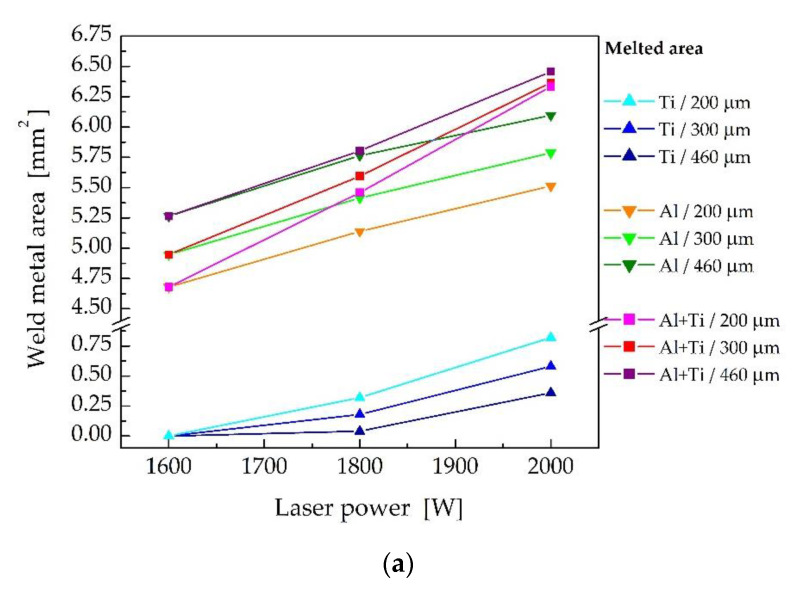

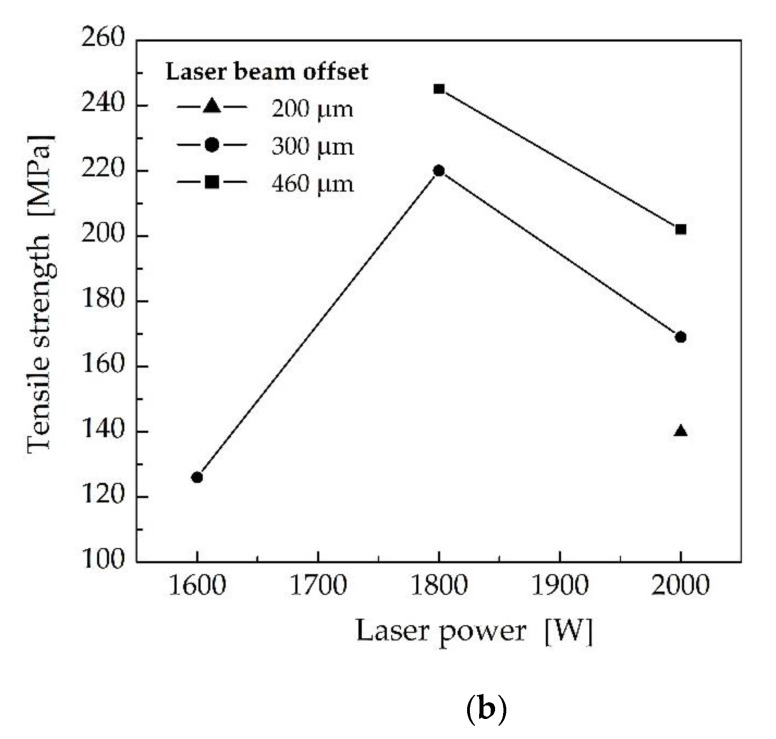
Effect of laser power (**a**) on the weld metal area and (**b**) on the ultimate tensile strength of the weld joints prepared with different laser beam offsets and a welding speed of 30 mm·s^−1^.

As the laser power increased, the size of the melted zones on both the Ti and Al sides increased for all considered laser beam offsets at a welding speed of 30 mm·s^−1^ (Figure 14a). When increasing the laser power from 1600 W to 1800 W, the ultimate tensile strength of the weld joints increased, but it decreased again for a laser power of 2000 W (Figure 14b). This decrease in ultimate tensile strength was related to the expansion of the melted zone on the Ti side.

**Figure 15:**
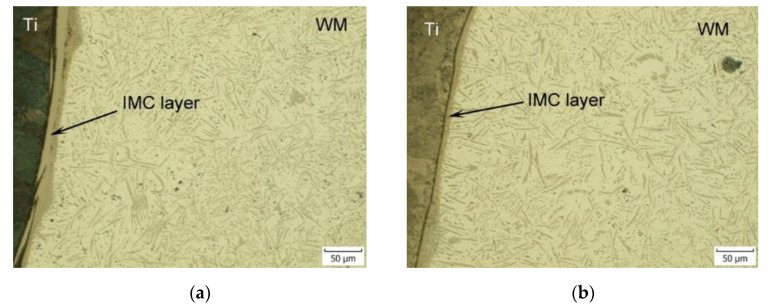
Ti Grade 2—weld metal interface for (**a**) sample 2 and (**b**) sample 4 produced at laser powers of 2000 W and 1800 W, respectively.

Figure 15 illustrates the details of the Ti Grade 2 and weld metal interface for samples 2 and 4 produced at laser powers of 2000 W and 1800 W. At a higher laser power, a larger amount of Ti Grade 2 melted, which promoted the diffusion of atoms at the interface between titanium and the weld metal and led to the formation of intermetallic phases. The thickness of the IMC layer at higher laser power was larger and uneven. The IMC interface layer at a laser power of 1800 W was continuous, and relatively narrow with a quasi-uniform thickness, resulting in the high tensile strength of this weld joint. 

The highest ultimate tensile strength was achieved for the weld joint prepared with a laser power of 1800 W, but having a larger offset of 460 μm, which could be attributed to reduced intermixing of the Ti and Al alloys and a further decrease in the IMC layer thickness. On the other hand, when no Ti melting occurred at a laser power of 1600 W, the measured values of the ultimate tensile strength of the welded joints were the lowest. Similar conclusions were reached by Sahul et al. [97], who additionally conducted detailed microstructural EDS and XRD analyses, microhardness testing, and fracture surface evaluation of produced Al/Ti weld joints. According to their observations, at a laser power of 1800 W, a welding speed of 30 mm·s^−1^ and a laser offset of 300 μm, the IMC layer consists of two zones: a thin continuous zone I directly on the Ti surface, with a thickness from 1.112 μm (at the top of the weld) to 1.238 μm (at the root of the weld), and an adjacent zone 2, whose thickness varied along the height of the weld from top to bottom, from 3.786 μm to 2.231 μm. Zone I was formed primarily of Ti_3_Al, while zone II contained 63.37 at.% aluminum, 35.81 at.% titanium and 1.12 at.% Mg, indicating the presence of TiAl_2_.

**Figure 16:**
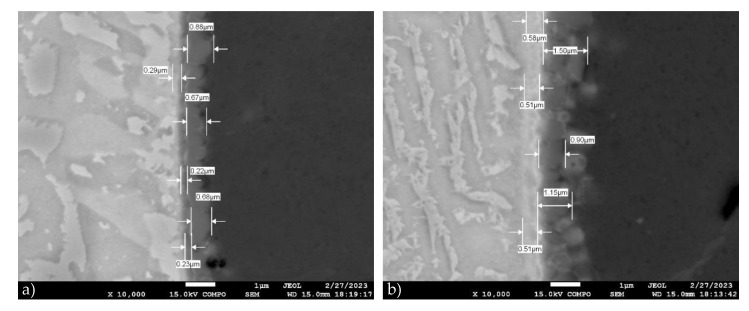
SEM images of Ti Grade 2—weld metal interface for sample 7 taken from (**a**) the top and (**b**) the root part of the welded–brazed joint (laser power of 1800 W, welding speed of 25 mm·s^−1^ and laser beam offset of 460 μm).

For comparison, SEM images of the Ti Grade2 weld metal interface of sample 7 are shown in Figure 16. When the laser beam displacement increased to 460 μm at a laser power of 1800 W, the thickness of zone I decreased to 0.533 ± 0.0404 μm at the top of the weld and to 0.246 ± 0.0378 μm in the middle part of the weld. Zone II had a thickness from 1.183 ± 0.3014 μm at the top of the weld to (0.743 ± 0.1185) μm in the middle of the weld. Increasing the laser offset from 300 μm to 460 μm at a laser power of 1800 W led to a three-fold reduction in the thickness of the discontinuous IMC layer (zone II). At the same time, the thickness of the continuous IMC layer on the Ti surface (zone I) was two times smaller in the upper part of the weld and approximately five times reduced in the middle part of the weld joint, leading to an increase in the tensile strength of the weld joint.

In conclusion, a low laser power of 1600 W did not cause melting of the Ti plate, resulting in low tensile strength of the weld joints. At a laser power of 1800 W, the melted area of Ti decreased from 0.32 mm^2^, for a laser beam offset of 200 μm, to 0.04 mm^2^ if the laser beam offset was enlarged to 460 μm. During rapid solidification of the melt, only a thin continuous IMC layer formed at the Ti–WM interface, which ensured the strength of the weld joint at a level of 220 MPa (for an offset of 300 μm) to 245 MPa (for an offset of 460 μm). A further increase in laser power to 2000 W led to an increase in the amount of melted titanium and, subsequently, to the formation of an IMC layer with a larger, and irregular, thickness, which was reflected in a decrease in the mechanical properties of the weld joint. 

### 5.3. Influence of the Welding Speed on the Temperature Fields

**Figure 17:**
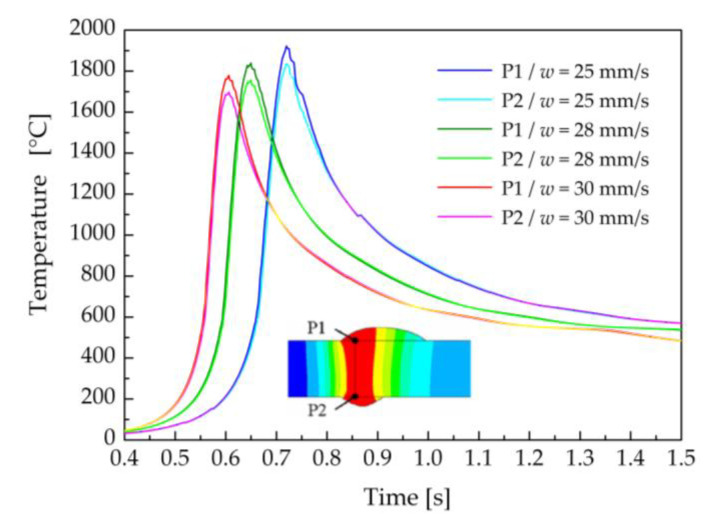
Time history of temperatures in the nodes P1 and P2 in the weld centreline for the welding speed of 25 mm·s^−1^, 28 mm·s^−1^ and 30 mm·s^−1^ (the laser power of 1800 W and the laser beam offset of 300 μm).

The influence of welding speed on the temperature fields during the welding–brazing process was evaluated at a constant laser power of 1800 W and a laser beam offset of 300 μm. The welding speed was changed from 25 mm·s^−1^ to 28 mm·s^−1^ and 30 mm·s^−1^. The effect of the welding speed on the maximum temperatures in the centerline of the weld on the top and bottom surfaces of the welded–brazed plates is illustrated in Figure 17. The maximum temperature in node P1 increased from 1778 °C to 1915 °C with decreasing welding speed from 30 mm·s^−1^ to 25 mm·s^−1^. This temperature increase was associated with the higher heat input used in the laser welding–brazing process at the lower welding speed.

**Figure 18:**
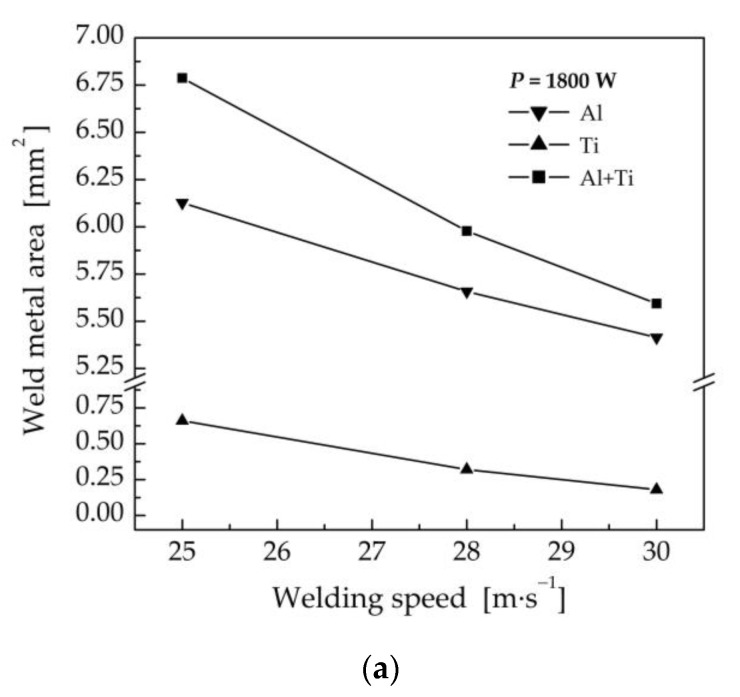

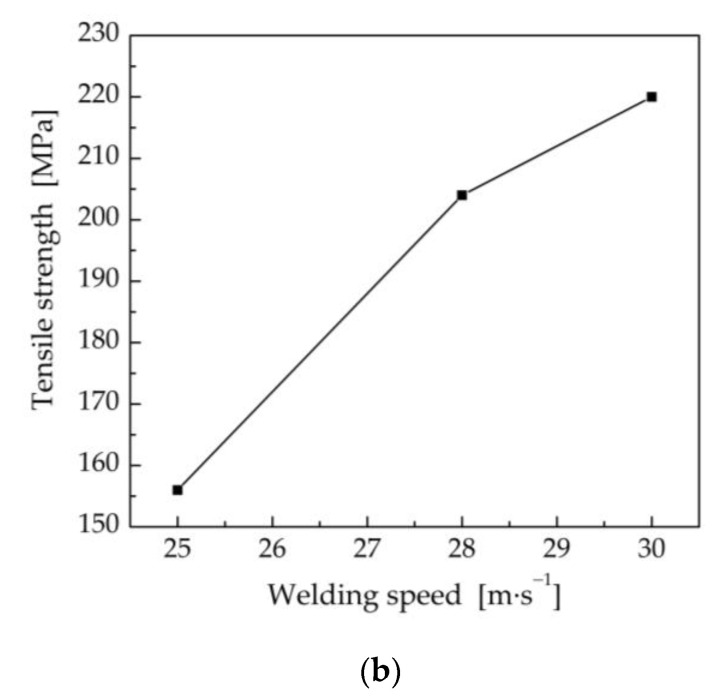
Effect of welding speed on the weld metal area and the ultimate tensile strength of the weld joints prepared with a laser power of 1800 W and a laser beam offset of 300 μm.

Due to the higher heat input at lower welding speeds, the weld metal area was also larger, as documented in Figure 18a. At the same time, the ultimate tensile strength decreased from 220 MPa, at a welding speed of 30 mm·s^−1^, to 156 MPa, for a welding speed of 25 mm·s^−1^ (Figure 18b). Based on previous findings on the relationship between the amount of melted titanium and the strength of the weld joint, higher welding speeds are recommended to prevent the melting of Ti Grade 2 and the formation of intermetallic compounds at the Ti–WM interface.

### 5.4. Prediction of Mechanical Properties of Welded–Brazed Joints

**Figure 19:**
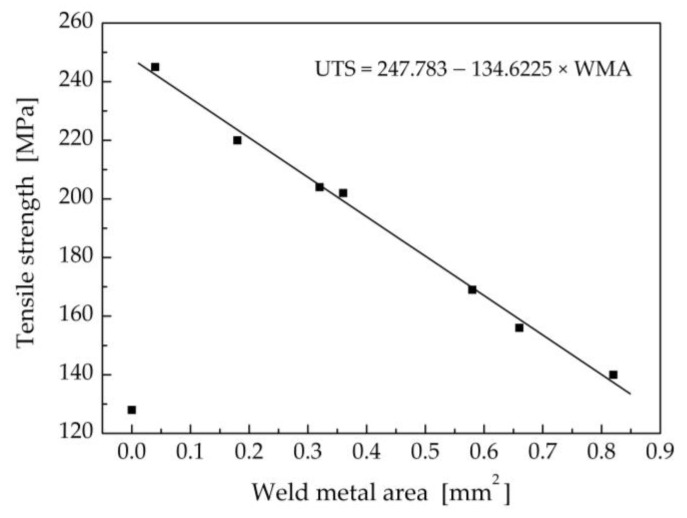
Dependence of the measured ultimate tensile strength of the weld joints on the computed Ti weld metal area.

The results achieved indicate that the ultimate tensile strength of the welded–brazed joints is mainly affected by the amount of melted Ti Grade 2. As shown in Figure 19, the measured values of the ultimate tensile strength decreased linearly with increase in the computed Ti weld metal area, regardless of the welding parameters, such as laser power, laser beam offset, and welding speed.

The ultimate tensile strength of a weld joint (UTS) can be approximately predicted using the computed cross-sectional area of melted Ti (WMA) from the function

UTS = 247.783 − 134.6225 × WMA for WMA > 0 [MPa]

## 6. Conclusions

The effect of welding parameters on the temperature fields generated during the laser welding–brazing of dissimilar Ti Grade 2 and EN AW5083-H111 aluminum alloy plates, using 5087 aluminum alloy filler wire, was investigated. The study was supported by numerical simulation of the laser joining process in the ANSYS program code. The heat supply to the weld joint was defined using a 3D conical heat source model. The developed simulation model was verified by comparing the results of numerical simulations to temperature cycles measured in the laser welding–brazing of experimental samples.

Based on the results of the numerical simulation, microstructural analysis, and tensile testing of the produced Al/Ti dissimilar welded–brazed joints it can be concluded that:

- The numerical simulation of the laser welding–brazing process was found to be sufficiently accurate and can be used to study the effects of welding parameters on the temperature fields and ultimate tensile strength of welded–brazed joints.
- The laser power has a significant impact on the temperature fields during the laser welding–brazing process, while the effect of the laser beam offset and welding speed are less pronounced.
- The ultimate tensile strength of the welded–brazed joints is primarily affected by the amount of melted Ti Grade 2. The thickness and morphology of the IMC layer that forms during rapid solidification of the melt are directly related to the amount of melted titanium.
- An increase in the laser beam offset towards the Al side leads to a decrease in the amount of melted Ti Grade 2, while the melted zone on the Al side increases, and, thus, resulting in an increase in the ultimate tensile strength of the welded–brazed joint.
- At a laser power of 1600 W, the fusion of Ti Grade 2 does not occur, resulting in low ultimate tensile strength of the weld joint at 135 MPa. As the laser power increases, the maximum temperatures of the weld pool also increase, causing a greater amount of melted titanium and the formation of an IMC layer at the Ti–WM interface. At a laser power of 1800 W, the melted area of Ti Grade 2 ranges from 0.32 mm^2^, for a laser beam offset of 200 μm, to 0.04 mm^2^, for a laser beam offset of 460 μm, leading to the maximum recorded ultimate tensile strength of 245 MPa. However, a further increase in laser power to 2000 W results in a higher amount of melted titanium and the formation of a wider and unevenly thick IMC layer, leading to a decrease in the mechanical properties of the weld joint.
- The increase in welding speed from 25 mm/s to 30 mm/s, at a laser power of 1800 W and a laser beam offset of 300 μm, leads to a decrease in the amount of melted Ti Grade 2, resulting in an improvement in the ultimate tensile strength of the welded–brazed joints. The tensile strength increased from 156 MPa to 220 MPa.
- The ultimate tensile strength (UTS) of Al/Ti dissimilar welded–brazed joints can be predicted, based on the computed cross-sectional Ti weld metal area (WMA), using the suggested formula UTS = 247.783 − 134.6225 × WMA [MPa].
- Taking into account the applied welding parameters to prepare Al/Ti welded–brazed samples 1–8, the highest ultimate tensile strength was achieved for samples 4 and 7, which were produced with a laser power of 1800 W, a welding speed of 30 mm·s^−1^, and laser beam offsets of 300 μm and 460 μm, respectively, resulting in a strength of 220 MPa and 245 MPa, respectively.

The maximum laser power of 1800 W, along with the welding speed of 30 mm·s^−1^ and the laser beam offset at least 300 μm towards the aluminum side can be recommended for the laser joining of Al/Ti dissimilar plates with a thickness of 2 mm. Higher values of laser power or decrease in welding speed lead to unfavorable melting of the Ti plate and formation of a brittle IMC layer.

## Figures and Tables

**Table 1:** Chemical composition of the EN AW5083-H111 aluminum alloy.

Element	Mg	Si	Fe	Mn	Cu	Cr	Al
**wt. %**	4.7	0.4	0.31	0.26	0.19	0.13	Balance

**Table 2:** Chemical composition of the titanium Grade 2.

Element	Fe	C	O	H	N	Ti
**wt. %**	0.3	0.1	0.25	0.015	0.03	Balance

**Table 3:** Chemical composition of the 5087 aluminum filler material.

Element	Si	Fe	Cu	Mn	Mg	Cr	Zn	Zr	Ti	Be	Al
**wt. %**	≤0.25	≤0.4	≤0.05	0.7–0.1	4.5–5.2	0.05–0.25	≤0.25	0.1–0.2	≤0.15	≤3 × 10^−4^	bal.

**Table 4:** Welding parameters applied for the experimental preparation of welded–brazed joints.

Sample No.	Laser Power [kW]	Welding Speed [mm/s]	Laser Beam Offset [μm]
1	2	30	460
2	2	30	300
3	2	30	200
4	1.8	30	300
5	1.8	28	300
6	1.8	25	300
7	1.8	30	460
8	1.6	30	300

## Data Availability

Not applicable.

## Nomenclature

**List of Symbols and Abbreviations**		
**Parameters**	**Unit**	**Definition**
*A*	[m^2^·s^−1^]	thermal diffusivity
*c*_p_	[J·kg^−1^·K^−1^]	specific heat
*G*	[m·s^−2^]	gravity acceleration
*H*	[W·m^−2^·K^−1^]	combined heat transfer coefficient
*h*_C_	[W·m^−2^·K^−1^]	convection heat transfer coefficient
*h*_R_	[W·m^−2^·K^−1^]	radiation heat transfer coefficient
*N*	[-]	parameter dependent of the value of Rayleigh number
*Off*	[μm]	laser beam offset
*q*_v_	[W·m^−3^]	volumetric density of internal heat sources
*r*_0_*, r*_e_, *r*_i_	[m]	the surface radii in the planes, *z* = *z*_e_ and *z* = *z*_i_, respectively (conical heat source model)
*T*	[s]	time
*W*	[mm·s^−1^]	welding speed
*x, y, z*	[-]	coordinates
*C*	[-]	parameter dependent of the value of Rayleigh number
*L*	[m]	characteristic length
*Nu*	[-]	Nusselt number
*P*	[W]	laser power
*Q*_0_	[W]	maximum heat source intensity
*Ry*	[-]	Rayleigh number
*T*(*x, y, z, t*)	[°C, K]	temperature
*T*_S_	[°C, K]	solidus temperature
*T*_L_	[°C, K]	liquidus temperature
*T*_0_	[°C, K]	initial temperature
*T*_w_	[°C, K]	surface/wall temperature
*T*_f_	[°C, K]	surrounding/fluid temperature
Δ*T*	[°C, K]	temperature difference between the surface temperature and surrounding temperature
*α*_v_	[K^−1^]	thermal expansion coefficient
*Ε*	[-]	emissivity
*λ*_x_, *λ*_y_, *λ*_z,_ *λ*_g_	[W·m^−1^·K^−1^]	thermal conductivities
*H*	[-]	efficiency
*Ν*	[m^2^·s^−1^]	kinetic viscosity
*Ρ*	[kg·m^−3^]	density
*σ*_0_	[W·m^−2^·K^−4^]	Stefan–Boltzmann constant
**Abbreviations**		
BPP	beam quality	
CMT	Cold Metal Transfer	
FE	finite element	
FSW	Friction Stir Welding	
IMC	intermetallic compound	
MIG	Metal Inert Gas	
SEM	Scanning Electron Microscopy	
TIG	Tungsten Inert Gas	
UTS	ultimate tensile strength	
WM	weld metal	
WMA	weld metal area	

## References

[1] Kalaiselvan K., Elango A. (2014). Laser beam welding of Ti/Al dissimilar thin sheets—A literature review. JAMME.

[2] Martinsen K., Hu S.J., Carlson B.E. (2015). Joining of dissimilar materials. CIRP. Ann.-Manuf. Techn.

[3] Möller F., Thomy C., Katayama S. (2013). Laser welding and brazing of dissimilar materials. Handbook of Laser Welding Technologies.

[4] Baqer Y.M., Ramesh S., Yusof F., Manladan S.M. (2018). Challenges and advances in laser welding of dissimilar light alloys: Al/Mg, Al/Ti, and Mg/Ti alloys. Int. J. Adv. Manuf. Technol..

[5] Zhang Y., Yu D., Zhou J.P., Sun D. (2021). A review of dissimilar welding for titanium alloys with light alloys. Metall. Res. Technol..

[6] Kuryntsev S. (2022). A Review: Laser Welding of Dissimilar Materials (Al/Fe, Al/Ti, Al/Cu)—Methods and Techniques, Microstructure and Properties. Materials.

[7] Mathers G. (2002). The Welding of Aluminium and Its Alloys.

[8] Leyens C., Peters M. (2006). Titanium and Titanium Alloys: Fundamentals and Applications.

[9] Sánchez Amaya J.M., Amaya-Vázquez M.R., Botana F.J. (2013). Laser welding of light metal alloys: Aluminium and titanium alloys. Handbook of Laser Welding Technologies.

[10] Casalino G., Mortello M., Peyre P. (2015). YAG laser offset welding of AA5754 and T40 butt joint. J. Mater. Process. Techn..

[11] Chen S., Li L., Chen Y., Huang J. (2011). Joining mechanism of Ti/Al dissimilar alloys during laser welding-brazing process. J. Alloys. Compd..

[12] Chen Y., Chen S., Li L. (2009). Effects of heat input on microstructure and mechanical property of Al/Ti joints by rectangular spot laser welding-brazing method. Int. J. Adv. Manuf. Technol..

[13] Möller F., Grden M., Thomy C., Vollertsen F. (2011). Combined Laser Beam Welding and Brazing Process for Aluminium Titanium Hybrid Structures. Phys. Procedia.

[14] Liedl G., Kratky A., Mayr M., Saliger A. Laser assisted joining of dissimilar materials. Proceedings of the IQCMEA-ICF-Processing, Performance and Failure Analysis of Engineering Materials.

[15] Chen S., Li L., Chen Y., Dai J., Huang J. (2011). Improving interfacial reaction nonhomogeneity during laser welding–brazing aluminum to titanium. Mater. Design..

[16] Tomashchuk I., Sallamand P., Méasson A., Cicala E., Duband M., Peyre P. (2017). Aluminum to titanium laser welding-brazing in V-shaped Groove. J. Mater. Process. Techn.

[17] Zhang Y., Huang J., Ye Z., Cheng Z. (2017). An investigation on butt joints of Ti6Al4V and 5A06 using MIG/TIG double-side arc welding-brazing. J. Manuf. Process.

[18] Song Z., Nakata K., Wu A., Liao J. (2013). Interfacial microstructure and mechanical property of Ti6Al4V/A6061 dissimilar joint by direct laser brazing without filler metal and groove. Mater. Sci. Eng. A.

[19] Chen X., Lei Z., Chen Y., Han Y., Jiang M., Tian Z., Bi J., Lin S., Jiang N. (2019). Effect of Laser Beam Oscillation on Laser Welding–Brazing of Ti/Al Dissimilar Metals. Materials.

[20] Zhang Y.F., Huang J.H., Ye Z., Cheng Z., Yang J., Chen S.H. (2018). Influence of welding parameters on the IMCs and the mechanical properties of Ti/Al butt joints welded by MIG/TIG double-sided arc welding-brazing. J. Alloy. Compd..

[21] Chen X., Lei Z., Chen Y., Han Y., Jiang M., Tian Z., Bi J., Lin S. (2020). Microstructure and tensile properties of Ti/Al dissimilar joint by laser welding-brazing at subatmospheric pressure. J. Manuf. Process.

[22] Sambasiva A.R., Madhusudhan G.R., Satya K.P. (2011). Microstructure and tensile properties of dissimilar metal gas tungsten arc welding of aluminium to titanium alloy. Mater. Sci. Techn. Ser..

[23] Li L., Zhao B., Wu X. (2019). Tungsten inert gas welding of dissimilar metals aluminum to titanium with aluminum based wire. Mater. Res. Express.

[24] Wei S., Li Y., Wang J., Liu K. (2014). Influence of Welding Heat Input on Microstructure of Ti/Al Joint During Pulsed Gas Metal Arc Welding. Mater. Manuf. Process..

[25] Cao R., Sun J.H., Chen J.H. (2013). Mechanisms of joining aluminium A6061-T6 and titanium Ti–6Al–4V alloys by cold metal transfer technology. Sci. Technol. Weld. Join..

[26] Majumdar B., Galun R., Weisheit A., Mordike B.L. (1997). Formation of a crack-free joint between Ti alloy and Al alloy by using a high-power CO_2_ laser. J. Mater. Sci..

[27] Tomashchuk I., Sallamand P., Cicala E., Patrice P., Grevey D. (2014). Direct keyhole laser welding of aluminum alloy AA5754 to titanium alloy Ti6Al4V. J. Mater. Process. Tech..

[28] Lee S.J., Takahashi M., Kawahito Y., Katayama S. (2015). Microstructural Evolution and Characteristics of Weld Fusion Zone in High Speed Dissimilar Welding of Ti and Al. Int. J. Prec. Eng. Man.

[29] Chen S.H., Li H., Chen L.Q. (2013). Interfacial reaction mode and its influence on tensile strength in laser joining Al alloy to Ti alloy. Mater. Sci. Technol..

[30] Lee S., Katayama S., Kim D.J. (2013). Microstructural behavior on weld fusion zone of Al-Ti and Ti-Al dissimilar lap welding using single-mode fiber laser. JKOSME.

[31] Węglowski M.St., Błacha S., Phillips A. (2016). Electron beam welding–Techniques and trends–Review. Vacuum.

[32] Zhang Y., Li Y., Luo Z. (2015). Microstructure and mechanical properties of Al/Ti joints welded by resistance spot welding. Sci. Technol. Weld. Join..

[33] Fuji A., Ikeuchi K., Sato Y.S., Kokawa H. (2004). Interlayer growth at interfaces of Ti/Al-1%Mn, Ti/Al-4.6%Mg and Ti/pure Al friction weld joints by post-weld heat treatment. Sci. Technol. Weld. Join..

[34] Kim Y.C., Fuji A. (2002). Factors dominating joint characteristics in Ti-Al friction welds. Sci. Technol. Weld. Join..

[35] Dressler U., Biallas G., Mercado U.A. (2009). Friction stir welding of titanium alloy TiAl6V4 to aluminium alloy AA2024-T3. Mater. Sci. Eng. A.

[36] Chen Y., Ni Q., Ke L. (2012). Interface characteristic of friction stir welding lap joints of Ti/Al dissimilar alloys. Trans. Nonferrous. Met. Soc. China.

[37] Song Z., Nakata K., Wu A., Liao J., Zhou L. (2014). Influence of probe offset distance on interfacial microstructure and mechanical properties of friction stir butt welded joint of Ti6Al4V and A6061 dissimilar alloys. Mater. Design.

[38] Chen Y.C., Nakata K. (2009). Microstructural characterization and mechanical properties in friction stir welding of aluminum and titanium dissimilar alloys. Mater. Design.

[39] Chen Y., Liu C., Liu G. (2011). Study on the Joining of Titanium and Aluminum Dissimilar Alloys by Friction Stir Welding. Open. Mater. Sci. J..

[40] Gurevich L.M., Trykov Y.P., Kiselev O.S. (2014). Formation of structural and mechanical inhomogeneities in explosion welding of aluminum to titanium. Weld. Int..

[41] Xia H., Wang S., Ben H. (2014). Microstructure and mechanical properties of Ti/Al explosive cladding. Mater. Design.

[42] Fronczek D.M., Chulist L., Litynska-Dobrzynska R., Szulc Z., Zieba P., Wojewoda-Budka J. (2016). Microstructure Changes and Phase Growth Occurring at the Interface of the Al/Ti Explosively Welded and Annealed Joints. J. Mater. Eng. Perform..

[43] Szachogluchowicz I., Sniezek L., Hutsaylyuk V. (2016). Low cycle fatigue properties of AA2519–Ti6Al4V laminate bonded by explosion welding. Eng. Fail. Anal..

[44] Ren J.W., Li Y.J., Feng T. (2002). Microstructure characteristics in the interface zone of Ti/Al diffusion bonding. Mater. Lett..

[45] Dheenadayalan K., Rajakumar S., Balasubramanian V. (2015). Effect of Diffusion Bonding Temperature on Mechanical and Microstructure Characteristics of Cp Titanium and High Strength Aluminium Dissimilar Joints. Appl. Mech. Mater..

[46] Rajakumar S., Balasubramanian V. (2016). Diffusion bonding of titanium and AA 7075 aluminum alloy dissimilar joints—Process modeling and optimization using desirability approach. Int. J. Adv. Manuf. Technol..

[47] Magin J., Balle F. (2014). Solid state joining of aluminum, titanium and their hybrids by ultrasonic torsion welding. Materialwiss. Werkst..

[48] Zhang C.Q., Robson J.D., Ciuca O., Prangnell P.B. (2014). Microstructural characterization and mechanical properties of high power ultrasonic spot welded aluminum alloy AA6111–TiAl6V4 dissimilar joints. Mater. Charact..

[49] Balle F., Magin J. (2014). Solid state joining of aluminium to titanium by high power ultrasonics. Mater. Sci. Forum.

[50] Zhang C. (2015). Ultrasonic Welding of Aluminium to Titanium: Microstructure, Properties, and Alloying Effects. PhD Thesis.

[51] Lv S.X., Jing X.J., Huang Y.X., Xu Y.Q., Zheng C.Q., Yang S.Q. (2012). Investigation on TIG arc welding-brazing of Ti/Al dissimilar alloys with Al based fillers. Sci. Technol. Weld. Join..

[52] Ma Z., Wang Ch., Yu H., Yan J., Shen H. (2013). The microstructure and mechanical properties of fluxless gas tungsten arc welding–brazing joints made between titanium and aluminum alloys. Mater. Design.

[53] Li U.K., Li Y., Wei S., Wang J. (2014). Interfacial Microstructural Characterization of Ti/Al Joints by Gas Tungsten Arc Welding. Mater. Manuf. Processes.

[54] Gao M., Chen C., Gu Y., Zeng X. (2014). Microstructure and Tensile Behavior of Laser Arc Hybrid Welded Dissimilar Al and Ti Alloys. Materials.

[55] Wang S.Q., Patel V.K., Bhole S.D., Wen G.D., Chen D.L. (2015). Microstructure and mechanical properties of ultrasonic spot welded Al/Ti alloy joints. Mater. Design.

[56] Ma Z., Zhao W., Yan J., Li D. (2011). Interfacial reaction of intermetallic compounds of ultrasonic-assisted brazed joints between dissimilar alloys of Ti-6Al-4V and Al-4Cu-1Mg. Ultrason. Sonochem..

[57] Chen X., Xie R., Lai Z., Liu L., Zou G., Yan J. (2016). Ultrasonic-assisted brazing of Al–Ti dissimilar alloy by a filler metal with a large semi-solid temperature range. Mater. Design.

[58] Tsirkas S.A., Papanikos P., Kermanidis T.H. (2003). Numerical simulation of the laser welding process in butt-joint specimens. J. Mater. Process. Tech..

[59] Kik T. (2020). Computational Techniques in Numerical Simulations of Arc and Laser Welding Processes. Materials.

[60] Mackwood A.P., Craferb R.C. (2005). Thermal modelling of laser welding and related processes a literature review. Opt. Laser. Technol.

[61] Lindgren L.E. (2006). Numerical modelling of welding. Comput. Method. App. M.

[62] Olabi A.G., Casalino G., Hashmi S. (2014). Mathematical Modeling of Weld Phenomena, Part 1: Finite-Element Modeling. Materials, Science, and Materials Engineering from Comprehensive Materials Processing.

[63] Casalino G., Mortello M. (2015). Modeling and experimental analysis of fiber laser offset welding of Al-Ti butt joints. Int. J. Adv. Manuf. Tech..

[64] Casalino G., Mortello M., Peyre P. (2016). FEM analysis of fiber laser welding of Titanium and Aluminum. Procedia CIRP.

[65] D’Ostuni S., Leo P., Casalino G. (2017). FEM Simulation of Dissimilar Aluminum Titanium Fiber Laser Welding Using 2D and 3D Gaussian Heat Sources. Metals.

[66] Behúlová M., Babalová E., Sahul M. (2017). Design of Laser Welding Parameters for Joining Ti Grade 2 and AW 5754 Aluminium Alloys Using Numerical Simulation. Adv. Mater. Sci. Eng..

[67] Behúlová M., Nagy M., Vrtiel Š. (2019). Prediction of temperature fields during laser welding of Al-Ti sheets using numerical simulation. AIP. Conf. Proc..

[68] Behúlová M., Babalová E., Nagy M. (2017). Simulation model of Al-Ti dissimilar laser welding-brazing and its experimental verification. IOP Conf. Ser. Mater. Sci. Eng..

[69] Zhan X., Bu H., Gao Q., Yan T., Ling W. (2019). Temperature field simulation and grain morphology on laser welding-brazing between Ti-6Al-4V and 1050 aluminum alloy. Mater. Res. Express.

[70] Guo S., Peng Y., Cui C., Gao Q., Zhou Q., Zhu J. (2018). Microstructure and mechanical characterization of re-melted Ti-6Al-4V and Al-Mg-Si alloys butt weld. Vacuum.

[71] Zhou X.F., Cao X.B., Zhang F., Duan J.A. (2022). Numerical and experimental investigation of thermal stress distribution in laser lap welding of Ti6Al4V and 2024 alloy plates. Int. J. Adv. Manuf. Technol..

[72] Duggirala A., Kalvettukaran P., Acherjee B., Mitra S. (2021). Numerical simulation of the temperature field, weld profile, and weld pool dynamics in laser welding of aluminium alloy. Optik.

[73] Li L., Gong J., Xia H., Peng G., Hao Y., Meng S., Wang J. (2021). Influence of scan paths on flow dynamics and weld formations during oscillating laser welding of 5A06 aluminum alloy. J. Mater. Res. Technol..

[74] Mooli H., Rao S.S., Satyanarayana G., Rao B.N. (2021). Numerical Simulations and Experimental Validation on LBW Bead Profiles of Ti-6Al-4V Alloy. Pertanika. J. Sci. Technol..

[75] Ai Y., Jiang P., Shao X., Li P., Wang Ch., Mi G., Geng S., Liu Y., Liu W. (2017). The prediction of the whole weld in fiber laser keyhole welding based on numerical simulation. Appl. Therm. Eng..

[76] Cho W., Na S., Thomy C., Vollertsen F. (2012). Numerical simulation of molten pool dynamics in high power disk laser welding. J. Mater. Process. Technol..

[77] Ai Y., Jiang P., Shao X., Li P., Wang Ch. (2017). A three-dimensional numerical simulation model for weld characteristics analysis in fiber laser keyhole welding. Int. J. Heat. Mass. Transf..

[78] Buttazzoni M., Zenz C., Otto A., Gómez Vázquez R., Liedl G., Arias J.L. (2021). A Numerical Investigation of Laser Beam Welding of Stainless Steel Sheets with a Gap. Appl. Sci.

[79] Ai Y., Jiang P., Shao X., Li P., Wang Ch., Mi G., Geng S., Liu Y., Liu W. (2022). The analysis of asymmetry characteristics during the fiber laser welding of dissimilar materials by numerical simulation. Int. J. Adv. Manuf. Technol..

[80] Esfahani M.R.N., Coupland J., Marimuthu S. (2015). Numerical simulation of alloy composition in dissimilar laser welding. J. Mater. Process. Technol..

[81] Halim S.B., Bannour S., Abderrazak K., Kriaa W., Autric M. (2021). Numerical analysis of intermetallic compounds formed during laser welding of Aluminum-Magnesium dissimilar couple. Therm. Sci. Eng. Prog..

[82] Zang C., Liu J., Tan C., Zhangb K., Song X., Chen B., Li L., Feng J. (2018). Laser conduction welding characteristics of dissimilar metals Mg/Ti with Al interlayer. J. Manuf. Proc..

[83] Faraji A.H., Maletta C., Barbieri G., Cognini F., Bruno L. (2021). Numerical modeling of fluid flow, heat, and mass transfer for similar and dissimilar laser welding of Ti-6Al-4V and Inconel 718. Int. J. Adv. Manuf. Technol..

[84] Ghosh P.S., Sen A., Chattopadhyaya S., Sharma S., Singh J., Dwivedi S.P., Saxena A., Khan A.M., Pimenov D.Y., Giasin K. (2021). Prediction of Transient Temperature Distributions for Laser Welding of Dissimilar Metals. Appl. Sci..

[85] Xie X., Zhou J., Long J. (2021). Numerical study on molten pool dynamics and solute distribution in laser deep penetration welding of steel and aluminum. Opt. Laser. Technol..

[86] Attar M.A., Ghoreishi M., Beiranvand Z.M. (2020). Prediction of weld geometry, temperature contour and strain distribution in disk laser welding of dissimilar joining between copper & 304 stainless steel. Optik.

[87] Huang W., Wang H., Rinker T., Tan W. (2020). Investigation of metal mixing in laser keyhole welding of dissimilar metals. Mater. Des..

[88] Liu J., Tan C., Wu L., Zhao X., Zhang Z., Chen B., Song X., Feng J. (2019). Butt laser welding-brazing of AZ31Mg alloy to Cu coated Ti-6Al-4V with AZ92 Mg based filler. Opt. Laser. Technol..

[89] Fotovvati B., Wayne S.F., Lewis G., Asadi E. (2018). A Review on Melt-Pool Characteristics in Laser Welding of Metals. Adv. Mater. Sci. Eng..

[90] Zhou X., Duan J., Zhang F., Zhong S. (2019). The study on mechanical strength of titanium-aluminum dissimilar butt joints by laser welding-brazing process. Materials.

[91] Jandaghi M.R., Saboori A., Khalaj G., Khanzadeh Ghareh Shiran M. (2020). Microstructural Evolutions and its Impact on the Corrosion Behaviour of Explosively Welded Al/Cu Bimetal. Metals.

[92] Shiran M.R.K.G., Bakhtiari H., Mousavi S.A.A.A., Khalaj G., Mirhashemi S.M. (2017). Effect of stand-off distance on the mechanical and metallurgical properties of explosively bonded 321 austenitic stainless steel-1230 aluminum alloy tubes. Mater. Res..

[93] Nagy M. (2020). Influence of technological parameters of laser welding on the microstructure and properties of aluminium and titanium weld joints. PhD Thesis.

[94] Kalaiselvan K., Sekar K., Elavarasi S. (2021). A Review on Process Parameters of Ti/Al Dissimilar Joint Using Laser Beam Welding. Int. J. Mech. Eng..

[95] Lindgren L.E. (2001). Finite element modelling and simulation of welding. Part 1: Increasing complexity. J. Therm. Stresses.

[96] Incropera P.F., De Witt D.P. (1996). Fundamentals of Heat and Mass Transfer.

[97] Sahul Mi., Sahul Ma., Vyskoč M., Čaplovič Ľ., Pašák M. (2017). Disk laser weld brazing of AW5083 aluminium alloy with titanium Grade 2. J. Mater. Eng. Perform..

[98] Ansys® Academic Research Mechanical, Release 18.2. https://www.ansys.com/academic/terms-and-conditions.

[99] (2012). JMatPro Help, Release 6.1, Sente Software Ltd. https://www.crz.gov.sk/data/att/265769_dokument1.PDF.

[100] Huy H., Argyropoulos S.A. (1996). Mathematical modelling of solidification and melting: A review, Modelling Simul. Mater. Sci. Eng.

[101] Contuzzi N., Campanelli S.L., Casalino G., Ludovico A.D. (2016). On the role of the Thermal Contact Conductance during the Friction Stir Welding of an AA5754H111 butt joint. Appl. Therm. Eng..

[102] Shanmugam N.S., Buvanashekaran G., Sankaranarayanasamy K., Manonmani K. (2009). Some studies on temperature profiles in AISI 304 stainless steel sheet during laser beam welding using FE simulation. Int. J. Adv. Manuf. Technol..

[103] Shanmugam N.S., Buvanashekaran G., Sankaranarayanasamy K. (2012). Some studies on weld bead geometries for laser spot welding process using finite element analysis. Mater. Design.

[104] Chukkan J.R., Vasudevan M., Muthukumaran S., Kumar R.R., Chandrasekhar N. (2015). Simulation of laser butt welding of AISI 316L stainless steel sheet using various heat sources and experimental validation. J. Mater. Process. Tech..

[105] Na S.-J., Cho W.I., Katayama S. (2013). Developments in modelling and simulation of laser and hybrid laser welding. Handbook of Laser Welding Technologies.

[106] Goldak J., Chakravariti A., Bibby M. (1984). A new finite element model for welding heat sources. Metall. Trans. B.

[107] Zain-Ul-Abdein M., Nélias D., Jullien J.F., Deloison D. (2008). Termo-mechanical Analysis of Laser Beam Welding of Thin Plate with Complex Boundary Conditions. Int. J. Mater. Form..

[108] Wu C.S., Wang G., Zhang Y.M. (2006). A new heat source model for keyhole plasma arc welding in FEM analysis of the temperature profile. Weld. J.

[109] Dal M., Fabbro R. (2016). An overview of the state of art in laser welding simulation. Opt. Laser. Technol..

[110] Casalino G., Mortello M. (2016). A FEM model to study the fiber laser welding of Ti6Al4V thin sheets. Int. J. Adv. Manuf. Tech.

[111] Ayoola W.A., Suder W.J., Williams S.W. (2017). Parameters controlling weld bead profile in conduction laser welding. J. Mater. Process. Techn..

[112] Casalino G., Hu S.J., Hou W. (2003). Deformation prediction and quality evaluation of the gas metal arc welding butt weld. Proceedings of the Institution of Mechanical Engineers, Part B. J. Eng. Manufacture.

[113] Kik T. (2020). Heat Source Models in Numerical Simulations of Laser Welding. Materials.

[114] Farias R.M., Teixeira P.R.F., Vilarinho L.O. (2022). Variable profile heat source models for numerical simulations of arc welding processes. Int. J. Therm. Sci..

[115] Chiocca A., Frendo F., Bertini L. (2019). Evaluation of Heat Sources for the Simulation of the Temperature Distribution in Gas Metal Arc Welded Joints. Metals.

